# Pemphigus vulgaris antigen mRNA quantification for the staging of sentinel lymph nodes in head and neck cancer

**DOI:** 10.1038/sj.bjc.6605470

**Published:** 2009-12-08

**Authors:** J Solassol, V Burcia, V Costes, J Lacombe, A Mange, E Barbotte, D de Verbizier, C Cartier, M Makeieff, L Crampette, N Boulle, T Maudelonde, B Guerrier, R Garrel

**Affiliations:** 1 Cellular Biology Department, Montpellier Teaching Hospital, Montpellier, France; 2 Head and Neck Surgery Department, Guy de Chauliac Hospital, Montpellier Teaching Hospital, Montpellier, France; 3 Pathology Department, Guy de Chauliac Hospital, Montpellier Teaching Hospital, Montpellier, France; 4 Medical Statistics Department, Guy de Chauliac Hospital, Montpellier Teaching Hospital, France; 5 Nuclear Medicine Department, Guy de Chauliac Hospital, Montpellier Teaching Hospital, Montpellier, France; 6 University of Montpellier-1, Montpellier, France

**Keywords:** diagnostic accuracy, Pemphigus vulgaris antigen, sentinel lymph nodes, immunohistochemistry, head and neck cancer

## Abstract

**Background:**

Molecular diagnosis has been proposed to enhance the intra-operative diagnosis of sentinel lymph node (SLN) invasion in head and neck squamous cell carcinoma (HNSCC). Although cytokeratin (CK) mRNA quantification with real-time reverse transcriptase-PCR (QRT–PCR) has produced encouraging results, the more discriminating markers remain to be identified.

**Methods:**

Pemphigus vulgaris antigen (PVA), squamous cell carcinoma antigen (SCCA), and CK17 mRNA were quantified using QRT–PCR, and the results were compared with an extensive histopathological examination of the entire SLNs on 78 SLNs harvested from 22 patients with HNSCC.

**Results:**

SCCA and CK17 quantification showed significantly higher mRNA values for macrometastases (MAs) than for either negative or isolated tumour cell (ITC) SLNs (*P*<0.01). Pemphigus vulgaris antigen allowed the discrimination of all MAs and micrometastases from both negative and ITC SLNs (*P*<0.001). For the neck staging of patients, considering metastatic *vs* non-metastatic status, receiver-operating characteristic curve analysis found areas under the curve of 93.8, 97.9, and 100% for CK17, SCCA, and PVA, respectively. With PVA, a cutoff value of 562 copies per 100 ng of cDNA permitted the correct distinction between patients with positive as opposed to negative neck nodes in all cases.

**Conclusion:**

PVA seems to be a highly promising marker for accurate intra-operative SLN staging in HNSCC by QRT–PCR.

In cN0 head and neck squamous cell carcinoma (HNSCC), use of the sentinel lymph node (SLN) technique has proved to be valuable in selecting pN+ patients for therapeutic neck dissection while sparing unneeded extended neck surgery for pN0 patients. However, the main pitfall of this strategy is that histopathological diagnosis based on serial sections with immunohistochemistry (SS-IHC) is far too time consuming to be made use of during surgery in routine practice (van Diest *et al*, 2001). A delayed pN diagnosis compels pN+ patients (up to 40% of cases) to undergo further additional surgery with an increased risk of post-operative complications and a damaged functional outcome (Kuntz and Weymuller, 1999; Schiefke *et al*, 2009). In this context, real-time reverse transcriptase-PCR (RT–PCR) may be helpful because it is operator independent and, with some adaptation, can be automated and rapidly performed (Raja *et al*, 2002; Hamakawa *et al*, 2004). In a previous study, we demonstrated that cytokeratin (CK)17 mRNA quantification could be evaluated in SLNs by semi-quantitative RT–PCR and that neck staging could be performed with relevant sensitivity and specificity when compared with SS-IHC staging (Garrel *et al*, 2006). However, minute micrometastases (MIs) sized <450 *μ*m produced signals similar to those of negative SLN controls and were thus undiagnosed by CK17 mRNA level quantification, showing the limitation of this mRNA marker for the detection of metastases in SLNs. Therefore, molecular test accuracy had to be improved so as to minimise the risk of ‘false-negative’ cases before proposing an effective clinical intra-operative application of such an approach. Recently, Pemphigus vulgaris antigen (PVA) and squamous cell carcinoma antigen (SCCA) have been highlighted as potential tumour-specific mRNA markers for the molecular staging of cervical lymph nodes in HNSCC (Ferris *et al*, 2005). However, to our knowledge, the reliability and accuracy of these markers has not been evaluated elsewhere for the molecular diagnosis of occult SLN metastases.

The aims of this study were (1) to develop sensitive and reproducible quantitative RT–PCR (QRT–PCR) assays for PVA, SCCA, and CK17 mRNAs for detecting and quantifying metastases in SLNs of HNSCC; (2) to investigate whether macrometastates (MAs) and MIs, including those sized <450 *μ*m, can be distinguished from isolated tumour cells (ITCs) and negative nodes using these assays; and (3) to identify the most accurate marker for molecular analysis of SLNs, compared with an extensive histopathological examination of serial sections.

## Materials and methods

### Demographic populations

Between March 2006 and December 2008, every patient seen at the Head and Neck department of the Montpellier Teaching Hospital with an untreated oral or oropharyngeal squamous cell carcinoma (cN0M0) was asked for their consent to be a participant in this study. The study received ethical approval from the clinical research board of the hospital. A total of 22 consecutive patients were included and none refused to participate. The clinical, demographic, and histopathological characteristics of the population are reported in Table 1. Inclusion criteria were the same as those described in a previous study (Garrel *et al*, 2005).

### SLN detection

Sentinel lymph nodes were detected using a *γ*-probe (X-PROBE Clerad-ARIES, Chatillon, France) after a peri-tumoural radiotracer injection (Nanocis, CIS BIO International, Saclay, France) and lymphoscintigraphy was performed on the day of surgery, as previously described (Garrel *et al*, 2005). Overall, 78 SLN samples were collected. The average number of SLNs per patient was 3.54 (range, 1–7) and the average number of SLNs per neck side was 2.44. Neck dissections were performed for all patients, according to the primary location for a total of 571 nodes.

### SLN histopathological workup

After excision, SLNs were split and each half was embedded with OCT, snap-frozen in pre-cooled isopentane, and stored at –80°C. Each frozen lymph node was sampled in its entirety without any paraffin embedding. Two 5-*μ*m-thick frozen slices were collected every 250 *μ*m under RNase-free conditions (one slide for haematoxylin–eosin (HE) stain and the other for pancytokeratin IHC). For example, a 1-cm node would be sectioned 2 × 40 times. The rest of the lymph node tissue was blended and sent for QRT–PCR analysis. Immunohistochemistry used primary antibodies anti-CKs AE1/AE3 (Novocastra Laboratories Ltd, Newcastle, UK) and was performed using the Ventana DAB Detection Kit with the BENCHMARK XT Ventana Medical Systems Automate (Illkirch, France).

### SLN and patient staging

Results were expressed according to Hermanek's classification, that is, ITCs <200 *μ*m, MI ⩽2 mm, and MA >2 mm (Hermanek *et al*, 1999). Of 78 SLN samples, 9 (11.54%) were positive for tumour, that is, containing at least 1 MI (*n*=5) or MA (*n*=4). The number of metastases per SLN was one each in five cases, and two, three, and four in one case each. Of the 78 SLN samples, 69 were negative for tumour, that is, containing ITC only (*n*=12) or no tumoural cell (*n*=57). Patient necks were staged by taking into account the maximum size of metastases found in their SLNs. Six patients (27.3%) were therefore staged SLN+ because of the presence of an MI or an MA in at least one of their SLNs. A total of 16 patients (72.7%) were staged SLN− because of the presence of ITC only (*n*=6) or because of the absence of any tumoural cells (*n*=10). Isolated tumour cells were not considered pathological, given that the clinical relevance of such a status is unknown. In all, 11 control lymph nodes from non-cancerous patients were analysed following the same procedure and were similarly entered for molecular analysis.

### Total RNA extraction and RT

Lymph node tissue remaining after tissue sectioning for immunohistochemical analysis was used for real-time RT–PCR quantification of target mRNA. For this, total RNA was extracted from frozen tissues using the RNeasy mini kit (Qiagen, Valencia, CA, USA) according to the manufacturer's instructions. RNA integrity was verified using a 2100 Bioanalyzer (Agilent Technologies, Santa Clara, CA, USA). For first-strand cDNA synthesis, 5 *μ*g of total RNA, 200 ng of random hexamers, and 1 *μ*l dNTP mix (10 mM) were incubated for 5 min at 65°C in a total volume of 13 *μ*l in RNAse-free water. A volume of 4 *μ*l of 5 × first-strand buffer, 1 *μ*l of 1 M DTT, 40 UI of RNAseOUT Recombinant Ribonuclease Inhibitor (Invitrogen, Carlsbad, CA, USA), and 200 Units of Superscript III (Invitrogen) were then added to make a total volume of 20 *μ*l. The mix was then incubated for 5 min at 25°C and thereafter for 60 min at 60°C. The reaction was stopped by incubating at 70°C for 15 min. Complementary DNAs were frozen at −20°C until use.

### Real-time QRT–PCR analysis

Gene-specific oligonucleotide primers, amplicon length, and PCR conditions for all primers tested are shown in Supplementary Table 1. PCRs were performed in 20 *μ*l final volumes in capillary tubes in a LightCycler instrument (Roche Diagnostic, Mannheim, Germany). Reaction mixtures contained 2 *μ*l of LightCycler FastStart DNA mastermix for SYBR Green I (Roche Diagnostic), 0.5 *μ*M of each primer, and 2 *μ*l of template DNA. All capillaries were sealed, centrifuged at 500 *g* for 5 s, and then amplified in a LightCycler instrument, with activation of polymerase (95°C for 10 min), followed by 45 cycles of 10 s at 95°C, 10 s at the annealing temperature, and 9 s at 72°C. The temperature transition rate was 20°C per s for all steps. The double-stranded PCR product was measured during the 72°C extension step by detection of fluorescence associated with the binding of SYBR Green I to the product. Fluorescence curves were analysed using LightCycler software version 3.5.

Data with regard to *CK17*, *PVA*, and *SCCA* were normalised according to data obtained from three housekeeping genes, including *β*_2_-*microglobulin* (*β*_2_-MG), *TBP*, and *RS9*. Only CK17, SCCA, and PVA mRNA quantification results normalised with TBP mRNA expression are presented in this paper, but similar results were found using the other two housekeeping genes (data not shown).

### Standard curves

Human *β*_2_-MG, RS9, TBP, CK17, PVA, and SCCA cDNAs were obtained from a normal lymph node, amplified with the indicated primers, and cloned into the pCR2.1 vector (TOPO TA Cloning Kit; Invitrogen). Plasmids were then digested with HindIII and XbaI restriction enzymes (Boehringer, Mannheim, Germany), extracted from 3% agarose gel, reamplified, and purified (PCR Purification Kit; Qiagen). The purified fragment solution was measured in a spectrophotometer, and the molecule number was calculated. Serial dilutions were then prepared, which ranged from 10^6^ to 10^1^ *β*_2_-MG, RS9, TBP, CK17, PVA, and SCCA molecules per 100 ng of cDNA in a background of herring sperm DNA in Tris-EDTA buffer, pH 8.0.

A calibration curve was generated by analysis of plasmid dilutions for each target. For this purpose, each calibrator was correlated to its threshold cycle value (Ct), that is, the cycle number when a given sample becomes positive, defined as a measured fluorescence >10 s.d. above the background fluorescence. Supplementary Figure 1 shows the amplification plot of the calibrators (Supplementary Figure 1A), the calibration curve (Supplementary Figure 1B), and the corresponding results when the melting curve was determined (Supplementary Figure 1C). For each analysis, concentrations of calibrators and samples were calculated from the calibration curve generated. The assay quantitatively detected 10 copies per 100 ng of cDNA for each target. Each measurement was performed in triplicate and was repeated at least twice. Thus, when the results obtained from 5 × 4 replicates of each calibrator and sample were considered, the intra-assay CV was <5%, whereas the inter-assay CV was <10%, as calculated by variation from the mean.

### Statistical analysis

A comparison of the different markers between each SLN group was carried out using a non-parametric Kruskal–Wallis test. Differences were considered statistically significant when *P*<0.05. Multivariate analysis was based on receiver-operating characteristic (ROC) curves, which allow the characterisation of the discrimination between two well-defined populations. Statistical analyses were performed using STATA 10.0 software (StataCorp 2007; Release 10, College Station, TX, USA) and mROC software (Kramar *et al*, 2001). Quantitative PCR data were analysed with the unpaired Mann–Whitney test using GraphPad InStat (version 3.06). The level of statistical significance was set at the value of *P*<0.05.

## Results

### Sensitivity of quantitative real-time RT–PCR assay

To estimate the sensitivity of QRT–PCR assays, serial dilution experiments were carried out for CK17, SCCA, and PVA. A volume of 100 ng of cDNA derived from a cDNA pool of three histologically positive SLNs was diluted from 10^1^ to 10^6^ and subjected to QRT–PCR assay. In Supplementary Figure 1A, amplification plots for the sample containing serial dilutions of cDNA derived from this pool are overlaid. Standard curves were constructed from plots for the calculated Ct values of each reaction. Threshold values obtained with the standard dilution series revealed quantification of CK17, SCCA, and PVA total RNA, with a linear range (*r*>0.99) from 10^1^ to 10^6^ copies per 100 ng cDNA in repeated experiments (Supplementary Figures 1A and B). All assays of a pool of positive SLN cDNAs ailed to generate results because of amplification of the processed pseudogenes, as observed with the melting curves (Supplementary Figure 1C). Three housekeeping genes, *RS9*, *TBP*, and *β*_2_-*MG*, were quantified as internal standards and similar results were obtained (data not shown).

### Correlation of SLN quantitative CK17, SCCA, and PVA mRNA expressions and histopathological findings

**Figure 1:**
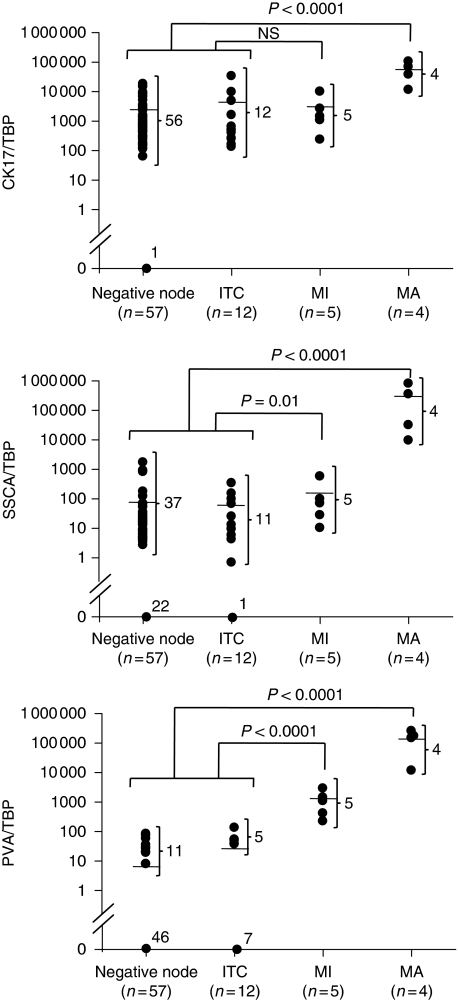
Absolute RT–PCR quantification of CK17, SCCA, and PVA mRNA in 78 SLNs. SLN status is determined according to histological status. mRNA quantification is expressed relative to the TBP housekeeping gene. Horizontal line, median. ITC, isolated tumour cell; MI, micrometastasis, MA, macrometastasis.

Among the 89 lymph nodes (78 SLNs and 11 control nodes), 88 (98.8%), 64 (72.7%), and 27 (30.3%) showed CK17 (3.1 × 10^5^ – 7 copies per 100 ng cDNA), SCCA (9.4 × 10^4^ – 2 copies per 100 ng cDNA), and PVA (6.4 × 10^4^ – 3 copies per 100 ng cDNA) mRNA expressions, respectively. In all, 3 of 4 MA and 0 of 5 MI SNLs expressed CK17/TBP mRNA values that were higher than the maximum value obtained for ITC (*n*=12) and the negative control (*n*=57). All MA SLN samples expressed higher SCCA/TBP mRNA values compared with those obtained from other groups. Interestingly, PVA/TBP mRNA values were higher for both MA and MI SNLs, including two MI SLNs sized <450 *μ*m, compared with the maximum mRNA value obtained for the negative-SLN group. To exclude false negatives, optimal threshold values were calculated to maximise sensitivity to 100% (CK17=236.6; SSC=9.9; PVA=179.8). Values above or equal to these ‘cutoff’ values were defined as QRT–PCR positive. The results of the comparison between histological examination and the absolute QRT–PCR method using a ‘cutoff’ value are shown in Table 2. No significant difference was found in the mRNA levels measured with QRT–PCR between the negative group (SLN without metastasis) and a group of 11 control LNs (non-cancerous patients) for CK17, SCCA, and PVA (*P*>0.05) (data not shown). The MA SLN group showed mean expression levels of CK17 that were higher than those of the negative control node group (negative node and ITC) (*P*<0.01), whereas MI SLNs expressed CK17 mRNA levels that were not significantly different from those of the negative control node group (Figure 1). Similar results were obtained for SCCA, with a significant difference observed only between the MA group and the negative node group (*P*<0.01) (Figure 1). However, interestingly, PVA showed a higher mRNA expression in the MA and MI SLN groups than in the negative control node group, with significant differences not only between MA (*P*<0.001) but also between MI SLN and controls (*P*<0.001) (Figure 1). None of the three tested markers allowed the ITC group to be distinguished from negative controls (*P*>0.05).

### Correlation of patient staging with quantitative CK17, SCCA, and PVA mRNA expression

**Figure 2:**
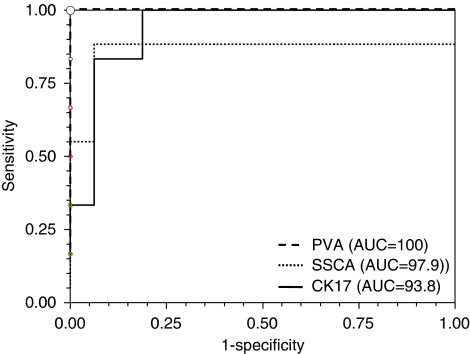
Receiver-operating characteristic curves for CK17, SCCA, and PVA. The AUC for PVA was 100%.

**Figure 3:**
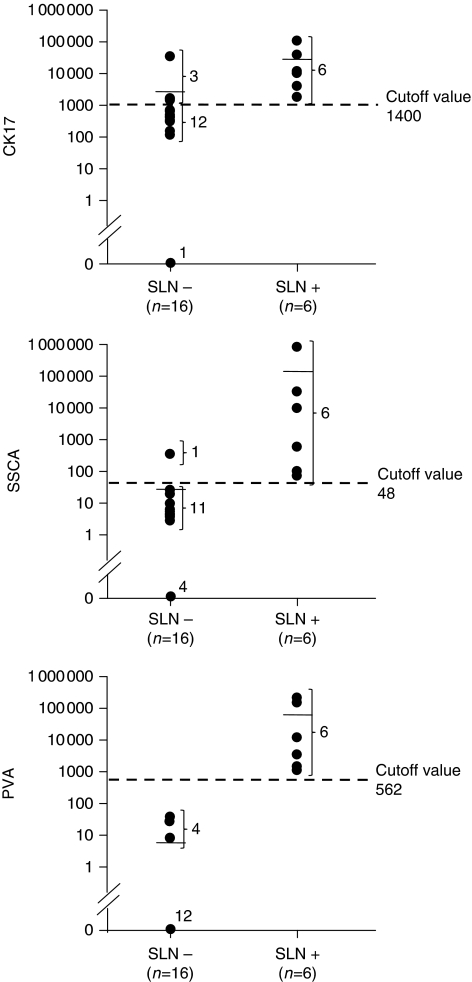
Patient staging: maximal expression of CK17, SCCA, and PVA for each individual patient as a function of serial step sectioning with immunohistochemistry. SLN, sentinel lymph node.

The CK17/TBP, SCCA/TBP, and PVA/TBP mRNA values were next used to analyse data according to patient status as determined by serial sections and IHC analyses (Table 2). A significant difference in mRNA levels in patients with metastasis-positive SLNs compared with metastasis-negative SLNs was found for CK17 (*P*<0.005), SCCA (*P*<0.0001), and PVA (*P*<0.0001). Receiver-operating curve analyses showed an area under the curve of 93.8% (95% CI (83.7–100)) for CK17, 97.9% (95% CI (92.8–100)) for SCCA, and 100.0% for PVA (Figure 2). Receiver-operating curve analyses for CK17 allowed the determination of a ‘cutoff’ value of 1436 with 81.2% specificity (95% CI (54.3–95.9)), 100% sensitivity (95% CI (54.1–100)), and 86.3% accuracy. For SCCA, a ‘cutoff’ value of 48 was calculated, with a specificity of 93.7 (95% CI (69.8–99.8)), a sensitivity of 100% (95% CI (54.1–100)), and an accuracy of 95.4. For PVA, 100% specificity (95% CI (79.4–100)), sensitivity (95% CI (54.1–100)), and accuracy were obtained with a ‘cutoff’ value of 562 (Figure 3). For an SLN invasion prevalence of 27.3%, the positive predictive values of CK17, SCCA, and PVA were 79.3, 91.2, and 100%, respectively.

## Discussion

At present, the missing link in the SLN strategy, particularly in HNSSC, seems to hinge on the accuracy of the intra-operative diagnosis of neck involvement. The SLN is defined as the first element that receives lymphatic drainage from an anatomical area harbouring a primary malignant tumour. Overall, 95% of cancer cells metastasise to SLNs (Zurrida *et al*, 2001). Sentinel lymph nodes are easily identified by injecting radioisotope or blue dye around the primary tumour. However, an important issue that needs to be solved in establishing the method is the diagnostic accuracy of MI. The worldwide ‘gold standard’ is HE staining. Lymph nodes are usually diagnosed as either metastatic or non-metastatic by examining one or two sections of the maximal cut surface. However, as the median size of the metastatic tissue to be diagnosed is ∼350 *μ*m, the sensitivity of routine frozen sectioning is only 20% (Atula *et al*, 2009). Semi-serial sectioning, associated with IHC, has a higher detection rate of MI than examination of only a few sections, and can reach the sensitivity of intra-operative fine-sectioned frozen sectioning of 93% (Tschopp *et al*, 2005). However, this approach is very time consuming and the inevitable loss of material can lead to some MIs being missed (van Diest *et al*, 2001). Furthermore, as the results reported are partly due to a high degree of attention and skill being provided by pathologists during clinical studies, such results are not likely to be achievable in a routine clinical setting (van Diest *et al*, 2001). Molecular diagnosis with QRT–PCR may be a substitute for histopathology for intra-operative SN analysis. Indeed, this rapid technique seems to be a valuable tool for the detection of lymph node invasion of breast adenocarcinomas (Kataoka *et al*, 2000), skin melanomas (Blaheta *et al*, 2001), and HNSCC (Hamakawa *et al*, 2000, 2004; Becker *et al*, 2004; Shores *et al*, 2004). We have previously shown that CK17 mRNA quantification could accurately determine sentinel node staging (Garrel *et al*, 2006). However, this marker did not permit the detection of MIs sized <450 *μ*m. As all undiagnosed MIs were associated with larger MIs or MAs, exact neck staging was finally achieved. Therefore, as the CK17 mRNA ‘cutoff’ value separating positive and negative nodes overlapped, more robust tumour-related markers clearly need to be validated. Recently, Ferris *et al* (2005) screened 40 potential markers using primary tumour and gross metastatic deposits and compared these with benign nodes. Among the screened markers, SCCA and PVA mRNA quantification provided a remarkable discrimination between positive and benign lymph nodes, and were thus proposed as potentially relevant markers for the staging of cervical lymph nodes in HNSCC. Despite this encouraging work, no studies have thus far addressed the possibility of intra-operative SCCA and PVA QRT–PCR analysis of SLNs in patients with HNSCC. We addressed this issue in this report and determined the diagnostic accuracy of the molecular diagnosis.

It is clear that real-time evaluation allows the detection of a signal at the earliest stage of metastasis, providing a more accurate indication of small tumour deposit than older RT–PCR methods that measure only at a defined end point. In this study, we first developed highly sensitive and quantitative assays for CK17, SCCA, and PVA RT–PCR assays (Supplementary Figure 1). We designed our original primers to minimise amplification of illegitimate mRNA. The expression level of each marker gene was calculated relative to the standard curve and corrected for the input of cDNA on the basis of the control housekeeping gene. Standard curves were generated from a pool of positive SLNs to ensure that every marker gene tested is expressed at high levels, thereby creating reproducible and reliable experiments. The results showed that very low levels of the marker-related gene (until 10^1^ copies per 100 ng cDNA for the three target genes, with *r*⩾0.98) could be detected. Second, we determined the reliability of real-time RT–PCR in comparison with histopathology data on 78 SLNs obtained from 22 HNSCC patients and 11 SLNs from non-cancerous patients. All MA SLNs were identified with SCCA and PVA mRNA quantification, whereas one of the four MA SLNs was not detected by real-time QRT–PCR of CK17 mRNA. In this latter case, the corresponding SLN was deemed positive by HE staining and IHC. In addition, PVA was the only marker that significantly distinguished small amounts of neoplastic tissue, that is, MI from negative nodes, including lesions <450 *μ*m. Interestingly, the background level and dispersion of mRNA values were very low for this marker (Figure 1). The QRT–PCR method was therefore used to establish a ‘cutoff’ value to maximise the sensitivity of the test (100%). Specificity increased progressively between CK17 and SCCA and reached 100% for SLN staging by PVA mRNA quantification (Table 2). With QRT–PCR, no false-negative case for PVA was observed in either SLN+ or SLN− patients, whereas one and three patients were misclassified with SCCA and CK17, respectively(Figure 3). Finally, mRNA quantification of SCCA and PVA allowed 95.4 and 100% accuracy, respectively. This important finding is in agreement with the study by Ferris *et al* (2005), which reported a test accuracy of 100 and 99.1%, respectively. By providing this 100% discrimination between positive and negative SLNs, PVA could be proposed as an adequate marker for the QRT–PCR diagnosis of minute SLN invasion of HNSCC.

With regard to the issue of ITCs, there is growing evidence that in head and neck cancer, ITC could be associated with a worse prognosis compared with pN0 SLN, a finding that contrasts with that which is observed in breast adenocarcinoma (Atula *et al*, 2009). Consequently, we assessed the ability of PVA mRNA quantification to diagnose ITC by varying the PVA cutoff point. Selecting a lower cutoff value of 22.68, only two patients with ITC were reclassified as ‘node-positive’, whereas four patients remained ‘node-negative’. Moreover, with this cutoff value, the specificity and sensibility rates of the test were 100 and 66.67%, respectively. Therefore, from these results, we concluded that, in our study, PVA is unable to distinguish ITC from ‘node-negative’ patients. The absence of any correlation between mRNA quantification and the presence of ITC can probably be explained by the histopathological workup of the lymph nodes used. The reference test, that is, serial sections every 250 *μ*m with IHC, is clearly not accurate enough to diagnose all cases of ITC, leading to some false-negative cases in the pN0 group. However, irrespective of the reason for these findings, a more focused study on ITC with a deeper histopathological workup and a more accurate quantification of minute tumours in SLN is highlighted as a useful area for future research.

With regard to markers, SCCA is a member of the ovalbumin family of serine proteinase inhibitors. The SCCA protein is expressed in neutral and acidic forms, designated as SCCA (SERPINB3) and SCCA2 (SEPINB4). Both genes locate at 18q21.3 very closely, generating a cluster of serpins, together with plasminogen activator inhibitor type 2 and maspin, suggesting that either the *SERPINB3* or the *SEPINB4* gene could arise from the other by gene duplication (Schneider *et al*, 1995). For SCCA amplification by RT–PCR, we designed primers that could detect both mRNA isoforms. Squamous cell carcinoma antigen-1 was originally purified from squamous cell carcinoma of the uterine cervix (Kato and Torigoe, 1977), and subsequently it turned out that SCCA1 and SCCA2 were co-expressed broadly in the superficial and intermediate layers of normal squamous epithelium. The biological functions of SSCA still remain obscure, although it has been reported that these proteins confer resistance against tumour necrosis factor-*α*- or radiation-inducing apoptosis (Suminami *et al*, 2000; Murakami *et al*, 2001). Finally, the expression of SCCA2 in cancer has been associated with an aggressive phenotype, and this gene has been used in several studies for the detection of squamous cell carcinoma metastases to lymph nodes (Kano *et al*, 2000). Pemphigus vulgaris antigen (also known as desmoglein 3 – DSG3) is one of the components of desmosome. Desmosomes are button-like points of intercellular contact that couple cytoskeletal elements to the plasma membrane at cell-to-cell or cell-to-substrate adhesions. A disorder related to DSG3 is pemphigus vulgaris (Amagai *et al*, 1991), an autoimmune disease associated with the production of autoantibodies against PVA responsible for the loss of cell-to-cell adhesion. Reports with regard to the clinical association of PVA expression with HNSCC cancer are few and seem inconsistent. Recently, Chung *et al* (2004) determined gene expression patterns from 60 HNSCC samples assayed on cDNA microarrays that allowed categorisation of these tumours with the clinical outcome of patients. Among the genes involved in the prognostic profile, PVA was found to be overexpressed in invasive cancer associated with a high metastatic potential. This result was confirmed more recently in a similar profiling approach (Chen *et al*, 2007). In our study, we observed that PVA was significantly expressed in SLN+ samples relative to SLN− samples. No cases with positive QRT–PCR and negative histopathology were observed. However, to avoid any such problem, careful dissection of the lymph node must be carried out so as to remove any contaminating non-lymphatic tissue. Finally, the morphological analysis of SS-IHC remains the gold standard for definitive histopathology to correct any erroneous diagnoses in cases of QRT–PCR inaccuracy and to search for histo-prognosis factors, such as capsular lymph node invasion. Therefore, if QRT–PCR analysis of SLNs is to be used in clinical practice, it should be included in multimodality diagnostic protocols similar to the one reported in this study.

We are aware that further investigations in larger cohorts are required to validate QRT–PCR of PVA mRNA and to establish inter-laboratory variations for an efficient detection, with high sensitivity and specificity, of metastatic disease in SLNs of patients with HNSCC. However, our findings have potentially important implications for the prospective assessment of molecular methodologies. We have provided additional data that could lead to better management of HNSCC patients by reducing the rate of false-negative SLNs using a QRT–PCR approach with quantification of PVA mRNA levels. On the basis of the present results, future studies assessing either an automated technique for mRNA quantification of PVA with QRT–PCR or using one-step nucleic acid amplification (Tsujimoto *et al*, 2007) may pave the way towards achieving worthwhile intra-operative molecular staging of SLNs in HNSCCs.

## Figures and Tables

**Table 1:** Clinicopathological characteristics of the HNSCC population

**Patient no.**	**Age (years)**	**Gender**	**Tumour location**	**cTNM**	**No. SLN**	**SLN location^a^**	**Histopathology of SLN^b^**	**Bilateral neck dissection**	**No. of non-SLN (neck dissection)**	**Final pN stage^c^**
1	29	M	OT	T_2_N_0_M_0_	4	IB, IIA	MI	No	29	pN_1_
2	58	M	OT	T_1_N_0_M_0_	3	III	MA	No	25	pN_1_
3	51	M	OC	T_1_N_0_M_0_	7	IB, II A, III	MI	Yes	27	pN_1_
4	53	M	OT	T_2_N_0_M_0_	5	IB, IIA, III	0	Yes	29	pN_0_
5	37	M	OT	T_2_N_0_M_0_	3	IB, IIA, IV	MA	No	18	pN_1_
6	44	M	OC	T_2_N_0_M_0_	4	IA,IB, III	MA	Yes	84	pN_2b_R+
7	55	M	OC	T_2_N_0_M_0_	3	IB, III	ITC	Yes	36	pN_0_
8	47	F	OT	T_1_N_0_M_0_	1	III	ITC	No	16	pN_0_
9	59	M	OP	T_2_N_0_M_0_	2	III	0	No	21	pN_0_
10	61	M	OC	T_2_N_0_M_0_	3	IB, IIA	0	Yes	40	pN_0_
11	57	M	OC	T_1_N_0_M_0_	5	IB, IIA, III, IV	0	Yes	36	pN_0_
12	31	F	OC	T_2_N_0_M_0_	4	IIA, IV	ITC	No	17	pN_0_
13	66	M	OT	T_1_N_0_M_0_	3	IB, IIA, III	ITC	No	17	pN_0_
14	49	F	OT	T_2_N_0_M_0_	3	III	0	Yes	8	pN_0_
15	79	M	OP	T_1_N_0_M_0_	4	IIA	0	No	3	pN_0_
16	75	F	OP	T_2_N_0_M_0_	4	II A,III	0	No	5	pN_0_
17	62	M	OT	T_2_N_0_M_0_	2	III	0	No	22	pN_0_
18	60	F	OC	T_1_N_0_M_0_	5	IA, IB, IV	0	Yes	13	pN_0_
19	61	M	OC	T_2_N_0_M_0_	3	IA, III	MI	Yes	44	pN2b
20	59	F	OP	T_2_N_0_M_0_	2	IIA, IIB	ITC	No	13	pN_0_
21	56	F	OP	T_1_N_0_M_0_	4	II A,III	0	No	17	pN_0_
22	49	M	OC	T_1_N_0_M_0_	4	IB, IIA, V	ITC	Yes	22	pN_0_

Abbreviations: cTNM=clinical tumour node-metastasis stage; FOM=floor of mouth; HNSCC=head and neck squamous cell carcinoma; ITC=isolated tumour cell; MA=macrometastasis; MI=micrometastasis; OP=oropharynx; OT=oral tongue; SLN=sentinel lymph node; SR=sex ratio; m=mean; T=total.

a Cervical lymph node levels according to Robbins *et al* (2002).

b Pathological examination of the SLN by serial step sectioning and immunohistochemistry according to the classification of Hermanek (6).

c pN stage was established according to the SLN and non-SLN status. The presence of isolated tumour cells was not taken into account in determining the final pN stage. No false negative cases were noted (pN+ with negative SLN)

**Table 2:** RT–PCR marker analysis according to lymph node staging (*n*=78) or patient staging (*n*=22)

	**SLN histological staging**				**Patient staging**	
	**Negative node (*n*=57)**	**ITC (*n*=12)**	**MI (*n*=5)**	**MA (*n*=4)**	**SLN− (*n*=16)**	**SLN+ (*n*=6)**
*CK17*						
Negative	9 (15.8%)	2 (16.6%)	0 (0%)	0 (0%)	13 (81%)	0 (0%)
Positive	48 (84.2%)	10 (83.3%)	5 (100%)	4 (100%)	3 (19%)	6 (100%)
						
*SSCA*						
Negative	36 (63.1%)	6 (50%)	0 (0%)	0 (0%)	15 (94%)	0 (0%)
Positive	21 (36.8%)	6 (50%)	5 (100%)	4 (100%)	1 (6%)	6 (100%)
						
*PVA*						
Negative	57/57 (100%)	12/12 (100%)	0 (0%)	0 (0%)	16 (100%)	0 (0%)
Positive	0 (0%)	0 (0%)	5 (100%)	4 (100%)	0 (0%)	6 (100%)

Abbreviations: CK17=cytokeratin 17; ITC=isolated tumour cell; MA=macrometastasis; MI=micrometastasis; RT–PCR=reverse transcriptase-PCR; SLN=sentinel lymph node; PVA=Pemphigus vulgaris antigen; SSCA=Squamous cell carcinoma antigen.
