# Supplementary figures and images for: Pemphigus vulgaris antigen mRNA quantification for the staging of sentinel lymph nodes in head and neck cancer
Source: Br J Cancer. 2009 Dec 8;102(1):181–7. doi: 10.1038/sj.bjc.6605470 (PMC2813735; doi:10.1038/sj.bjc.6605470)

## Slide 1
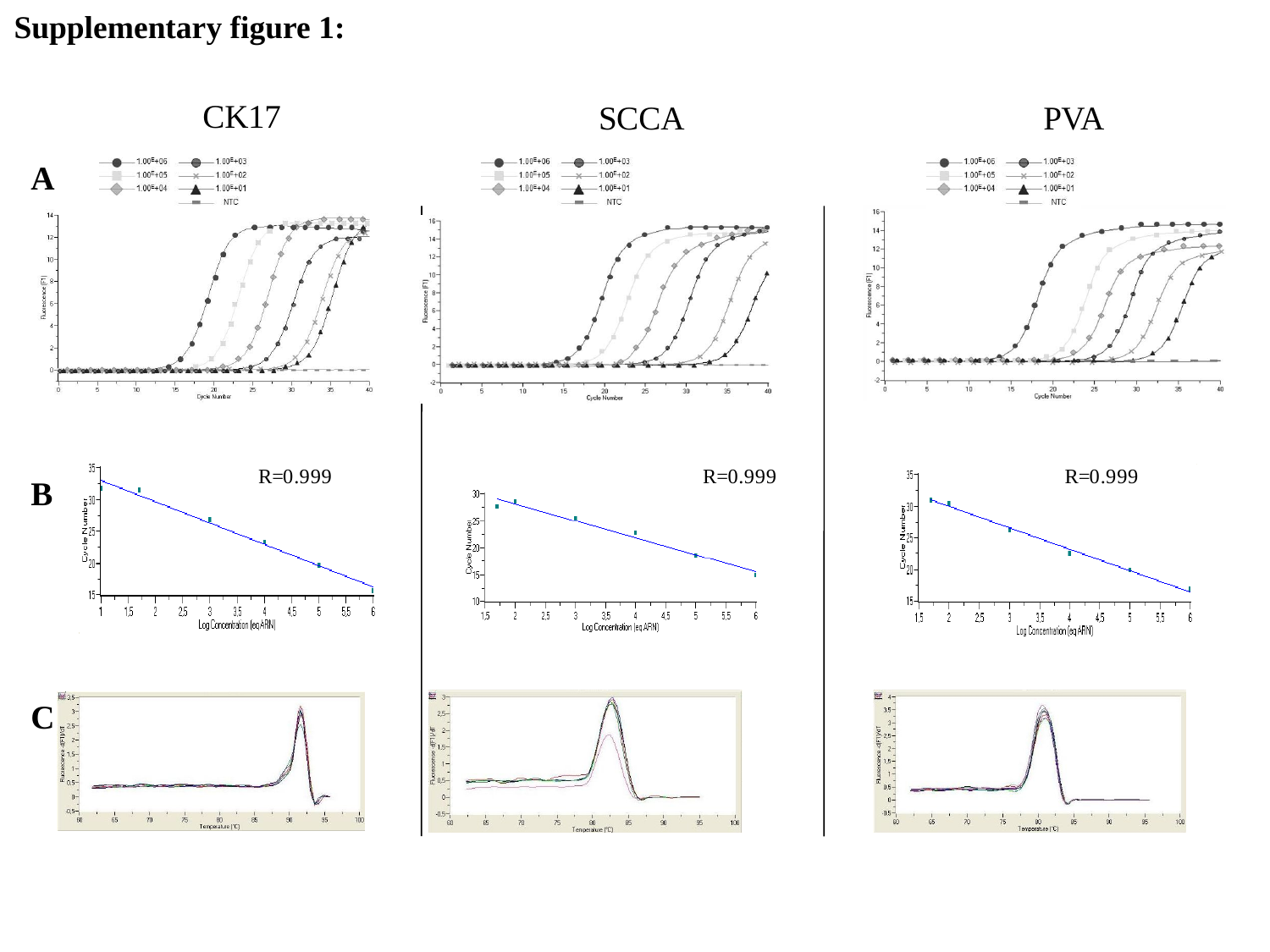

Supplementary figure 1:

Supplement: Supplementary Figure 1 [file 6605470x1.ppt]
