# Supplementary material for: Pemphigus vulgaris antigen mRNA quantification for the staging of sentinel lymph nodes in head and neck cancer
Source: Br J Cancer. 2009 Dec 8;102(1):181–7. doi: 10.1038/sj.bjc.6605470 (PMC2813735; doi:10.1038/sj.bjc.6605470)
Supplement: Supplementary Figure Legend [file 6605470x2.doc]

**Supplementary figure 1:** CK17, PVA, and SCCA series. (A) Fluorescence data from CK17, PVA, and SCCA standard dilution series. The x-axis provides the number of PCR cycles, whilst the y-axis shows the relative change in fluorescence intensity during PCR cycling. Each symbol represents the indicated absolute number of starting templates per 100 ng of cDNA. (B) Standard curve of CK17, PVA, and SCCA. X-axis: absolute copy number of starting template; Y-axis: mean threshold cycle (ct) of repetitive analyses of CK17, PVA, and SCCA standards. (C) CK17, PVA, and SCCA standard dilution series amplified by conventional RT-PCR. Bands in a 3% agarose gel after electrophoresis and ethidium bromide staining shown are: MW_1_, 25 bp ladder; MW_2_, 123 bp ladder; NTC, non-template control.
