# Supplementary material for: Pemphigus vulgaris antigen mRNA quantification for the staging of sentinel lymph nodes in head and neck cancer
Source: Br J Cancer. 2009 Dec 8;102(1):181–7. doi: 10.1038/sj.bjc.6605470 (PMC2813735; doi:10.1038/sj.bjc.6605470)
Supplement: Supplementary Table [file 6605470x3.doc]

**Table 1 supplemental:** Primer sequences used in QRT-PCR assays

| **Name** | **Sequence** | **Annealing temperature** |
| --- | --- | --- |
| β2-MG forward | 5' ACT ACA CTG AAT TCA CCC CC 3' | 60°C |
| β2-MG reverse | 5' TTA AAA AGC AAG CAA GCA GA 3' | 60°C |
| RS9 forward | 5' AAG GCC GCC CGG GAA CTG CTG AC 3' | 60°C |
| RS9 reverse | 5' ACC ACC TGC TTG CGG ACC CTG ATA 3' | 60°C |
| TBP forward | 5' CAC GAA CCA CGG CAC TGA TT 3' | 60°C |
| TBP reverse | 5' TTT TCT TGC TGC CAG TCT GGA 3' | 60°C |
| CK17 forward | 5' GGC CCG CCC GTG ACT ACA 3' | 62°C |
| CK17 reverse | 5' ATC TCC TCC TCG TGG TTC TTC TTC 3' | 62°C |
| PVA forward | 5' CAT GAA TTT TCT GGA CTC CTA CTT TTC T 3’ | 62°C |
| PVA reverse | 5' TTG CTT CCT GGC CAT CGT 3' | 62°C |
| SCCA forward | 5' CTG CCA AAT GAA ATC GAT GGT 3' | 62°C |
| SCCA reverse | 5' GCA AAC TTG TCC ATT CCA TCA A 3' | 62°C |
